# Using Occlusion-Based Saliency Maps to Explain an Artificial Intelligence Tool in Lung Cancer Screening: Agreement Between Radiologists, Labels, and Visual Prompts

**DOI:** 10.1007/s10278-022-00631-w

**Published:** 2022-04-28

**Authors:** Ziba Gandomkar, Pek Lan Khong, Amanda Punch, Sarah Lewis

**Affiliations:** 1 Discipline of Medical Imaging Science, Faculty of Medicine and Health, University of Sydney, Sydney, NSW Australia; 2 Clinical Imaging Research Center (CIRC), Department of Diagnostic Radiology, Yong Loo Lin School of Medicine, National University of Singapore, Singapore, Singapore

**Keywords:** Artificial intelligence, Occlusion-based saliency maps, Lung computed tomography, Radiologists

## Abstract

Occlusion-based saliency maps (OBSMs) are one of the approaches for interpreting decision-making process of an artificial intelligence (AI) system. This study explores the agreement among text responses from a cohort of radiologists to describe diagnostically relevant areas on low-dose CT (LDCT) images. It also explores if radiologists’ descriptions of cases misclassified by the AI provide a rationale for ruling out the AI’s output. The OBSM indicating the importance of different pixels on the final decision made by an AI were generated for 10 benign cases (3 misclassified by the AI tool as malignant) and 10 malignant cases (2 misclassified by the AI tool as benign). Thirty-six radiologists were asked to use radiological vocabulary, typical to reporting LDCT scans, to describe the mapped regions of interest (ROI). The radiologists’ annotations were then grouped by using a clustering-based technique. Topics were extracted from the annotations and for each ROI, a percentage of annotations containing each topic were found. Radiologists annotated 17 and 24 unique ROIs on benign and malignant cases, respectively. Agreement on the main label (e.g., “vessel,” “nodule”) by radiologists was only seen in only in 12% of all areas (5/41 ROI). Topic analyses identified six descriptors which are commonly associated with a lower malignancy likelihood. Eight common topics related to a higher malignancy likelihood were also determined. Occlusion-based saliency maps were used to explain an AI decision-making process to radiologists, who in turn have provided insight into the level of agreement between the AI’s decision and radiological lexicon.

## Introduction

Lung cancer is one of the leading causes of cancer-related deaths [1]. Worldwide, trials for the effectiveness of low-dose computed tomography (LDCT) scanning have been undertaken, with clear gains in cancer detection at earlier stages and subsequent improved treatment options and morality [2]. The cost-effectiveness of the lung cancer screening is one of the major implementation challenges, given its intensive visual task. Using artificial intelligence (AI) as a tool to aide radiologists might potentially increase the number of detected cancers and hence improve the cost–benefit balance of lung cancer screening [3, 4].

There have been many small studies incorporating AI into the screening process with very promising results [3–11]; however, no country is at the stage where guidelines for LDCT lung cancer screening incorporating AI have been produced and it is unknown how radiologists interact with AI at the level of decision-making. Different scenarios [12] for a radiologist-in-the-loop model could be envisaged and it is unknown how AI and radiologists can operate in a complementary way to produce the most efficient screening outcomes in the context of lung cancer screening. More efficient use of AI tools requires an improved computer–human interface so that radiologists can understand AIs and place the appropriate level of trust.

There are different possible options to present the AI prompts to the radiologists and the optimal prompting strategy is unknown. The output of the AI tools can be presented in various forms to the radiologists; for example, all possible suspicious areas can be visually shown, or the radiologist can click on any area of interest on an image and get the AI feedback. Other options include allowing the user to click on the AI prompt to launch a pop-up menu to see confidence scores, AI generated text description of the identified lesions using radiological lexicon, etc. Moreover, interpretability of the model, which refers to providing an understanding of algorithm output to the end-user, could help the radiologist to garner trust in a deployed AI.

Most of the medical imaging studies, aiming at making an AI tool interpretable [13], have used a visualization technique to produce a map overlaid on the original medical image to indicate the areas on which AI relied for making the final diagnosis/decision [14]. To model interpretation, the most direct approach is visualizing the network’s hidden layers, through inspection of the learned filters and feature maps. As example, Molle et al. [15] used feature maps and suggested that the high-level convolutional layers activate on similar concepts as utilized by human experts, such as lesion margin, presence of darker regions within the lesion, or appearance of the surrounding skin. Another group of visualization algorithms are perturbation-based methods, which rely on altering a part of image (e.g., by occluding parts of an image or adding noise) and monitoring how strongly those perturbations affect the model’s output. Kermany et al. [16] performed an occlusion testing on a dataset of optical coherence tomography images to identify the areas contributing most to the AI’s diagnosis. When verified by human experts, it was shown that nearly in 95% of images, such testing successfully identified the most clinically significant areas of pathology. Uzunova et al. [17] used occlusion-based maps to show the diagnostically relevant areas on classification neural network on optical coherence tomography images. The usefulness of this approach with brain MRI [18] and histopathological images [19] was explored. Many other visualization techniques such as guided backpropagation [20] and class activation mapping [21] have been proposed in the past few years to interpret AI models.

Although many visualization techniques are pseudo-validated by expert human observers in a sense that they are primarily through the highlighted regions only that were considered were diagnostically relevant, it is unknown that if presenting these maps overlaid on the original medical images without further medical annotation or labeling of pathologies would facilitate effective trust calibration in AI by radiologists. It is also unknown what the level of agreement by radiologists may be when that area is cued or highlighted by a method of visualization.

In this study, we explored whether only by showing the regions on which AI relied upon to make decisions, radiologists would agree on a high-level medical label of the area (e.g., differentiating a nodule from an artery/vein/vessel). We also explored if by only by pointing to the nodular areas, the radiologists would agree on radiological descriptors for explaining the nodule’s features. We categorized these radiological features as “topics” suggestive of higher or lower likelihood of malignancy and investigated the level of agreement among radiologists in selecting these two sets of topics for benign and malignant nodules. We included nodules, both correctly classified and misclassified by the AI, and explored if the level of agreement differed between these two groups. As the focus of this study is the observer agreement, we used a previously developed deep learning model for lung CT interpretation [3] and used the occlusion-based technique suggested in [22] to produce the saliency maps. No matter what visualizing technique is used, currently, the models show the areas significant to AI’s decision (sometimes with a confidence level presented in a format of text or in a format of colormap) to the radiologists. The tools usually do not provide any further labels describing what actually this area is. Lack of agreement among radiologists in recognizing or describing the areas would suggest radiologists may require further information about the highlighted areas to understand AI’s decision and only indicating diagnostically relevant areas are not sufficient to trust AI’s decisions or rule out its output.

## Materials and Methods

### Experimental Design

This study was approved by the Human Research Ethics Committee of the University of Sydney (Project Number 197/2019). We deployed a previously developed deep learning model for lung cancer detection [3]. A blinded test set of LDCT scans were fed into the trained model and using the occlusion-based technique suggested in [22], the saliency maps were generated. The occlusion-based method for unboxing an AI tool is a form of perturbation-based visualization technique [23]. The occlusion-based saliency maps indicate the importance of different pixels on the final decision of AI. In medical imaging, studies on brain MRI [17, 18, 24] and studies involving optical coherence tomography images, histopathological images [19], and chest X-rays [25] have shown the effectiveness of this approach to identify regions of importance. One of the main drawbacks of this approach is its computational burden; as for producing a high-resolution saliency map, the inference from the network must be performed many times. In our case, since we did this experiment on 20 images derived from 20 cases, using this approach was feasible. OBSMs have been recently used by Venugopal et al. [26] to unbox AI tool for lung CT images as well. Briefly, to produce the occlusion-based saliency, various regions of the image was systematically (i.e., with a pre-defined with a stride) masked and the changes in the malignancy score were recorded. Here we used a patch size of 4 × 4 × 4 to block the image, as suggested in [26]. For example, for an input image of 128 × 128 × 128 and a patch size of 4 × 4 × 4 to block the image with a stride of 4 steps per block, an output saliency map of 32 × 32 × 32 was acquired. The output saliency maps were then zoomed back to the original image resolution to form and create the final image, which was overlaid onto the original image. The test set included 10 benign cases (3 misclassified by the AI as malignant) and 10 malignant cases (2 misclassified by the AI as benign).

**Fig. 1:**
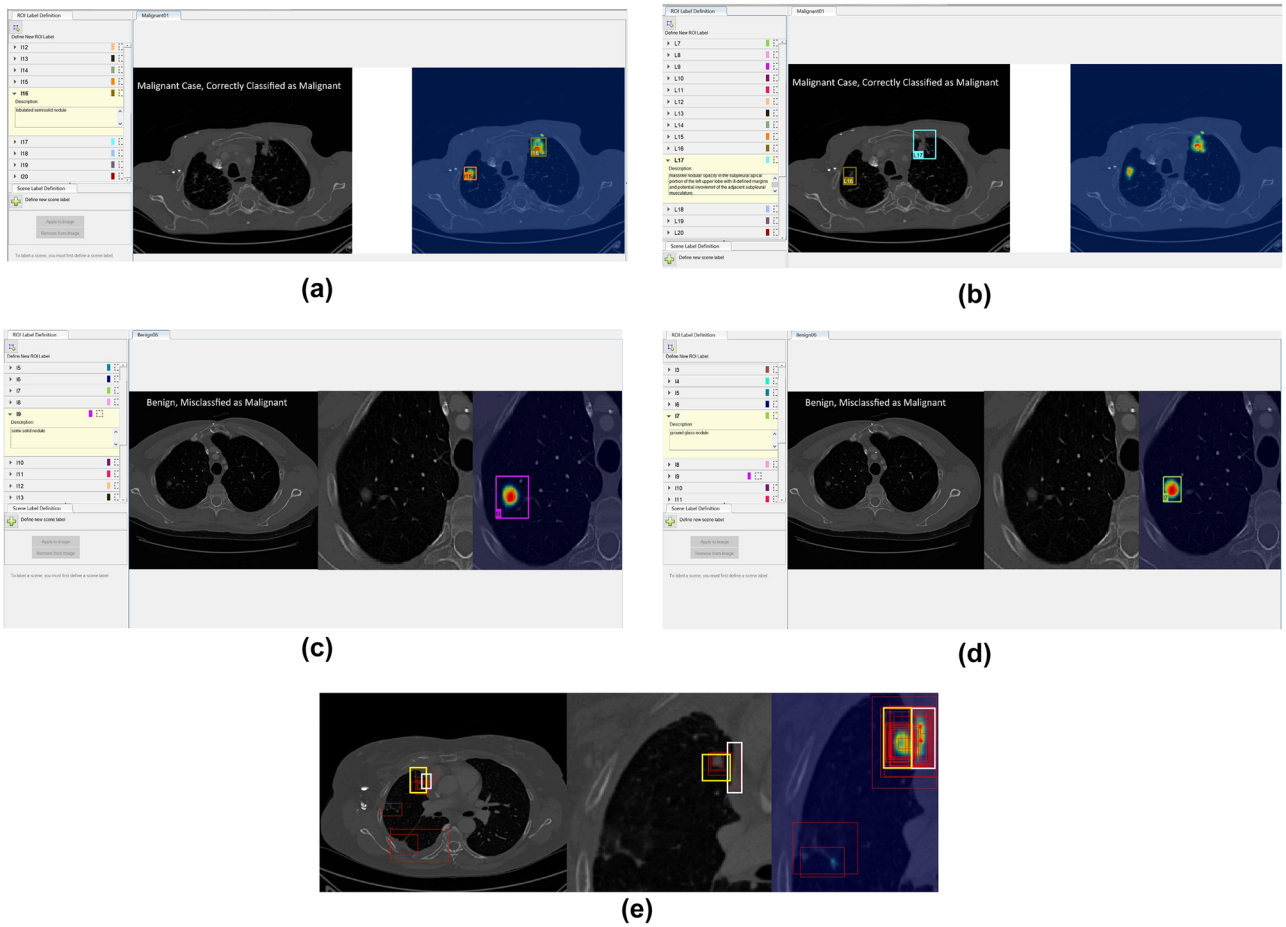
The environment for annotating the images. The case presented in (**a**) and (**b**) is a malignant case, correctly classified as malignant by the AI. Examples of the annotations provided by two radiologists to the same case are shown. Radiologists were asked to provide descriptors using the lexicon they usually use to report on lung CT images. In some cases (similar to a), only a few descriptors were assigned to a case while in other cases (similar to b), more comprehensive set of textual descriptors were provided. An example of misclassified benign and textual descriptors provided by two radiologists is shown in (**c**) and (**d**). In (**e**), one of the cases with annotations from all participants and the clustering results is shown

The original images and the corresponding saliency maps were presented to the radiologists using a MATLAB-based application. In the software application for annotating, the participants could draw rectangular areas. To draw one rectangular area, the participant had to start by clicking in the location to set a starting point (upper left corner of the rectangle). Then, they had to keep their mouse button held down and drag diagonally to draw the rest of the rectangle. As they dragged, they would have seen a thin outline of what the enclosed area would look like. The participants were asked to use their radiological vocabulary (such as terms they would use in a general radiology report when viewing LDCT for cancer screening) to describe the anatomical saliency mapped regions of interest (ROI). We deliberately zoomed on the ROI that contained the activated area on the OBSM, selecting the CT slice with the highest intensity level on the saliency map. Zooming and panning options were available on the original, zoomed, and OBSM images. An example of image with annotations provided by two radiologists is shown in Fig. 1. As shown, the radiologists were allowed to annotate the ROI on any of the image they preferred or considered important in classifying the image. We provided instructions to the radiologists to interpret the colors (ranging from blue to red, showing the low to high probability of abnormality). On the left side panel, the textual descriptors provided by the radiologists are shown. As shown in Fig. 1a, a participant might assign only a few descriptors to a case while in other occasions, as indicated in Fig. 1b, more comprehensive set of textual descriptors could be provided. An example of a misclassified benign image, and the corresponding textual descriptor provided by two radiologists, is also shown in Fig. 1c, d. Radiologists were allowed to annotate the areas shown in the heat-mapped ROI either on the OBSM or on the original image. Therefore, saliency maps and original images were registered on each other and clusters corresponding to similar image areas were combined. In Fig. 1e, correspondence between the areas for white and yellow clusters on three images is shown.

### Participants

Radiologists were recruited at the Radiological Society of North America (RSNA) Perception Lab event in December 2019, a dedicated area of the scientific meeting that offers delegates the opportunity to participate in research experiments that can be conducted within short timeframes. Based on their responses to our questionnaire, recruited participants were all registered radiologists in their home nations and had on average 11.5 ± 10.4 years of experience as a radiologist (range: 1 to 42 years) and 10.2 ± 9.8 years of experience in reading lung CT images (range: 1 to 42 years). On average, the participants spent 9.6 ± 10.2 h (range: > 1 to 20 h) interpreting lung CT images per week. Informed consent was obtained from all individual participants included in the study.

### Analysis of the Annotations

**Fig. 2:**
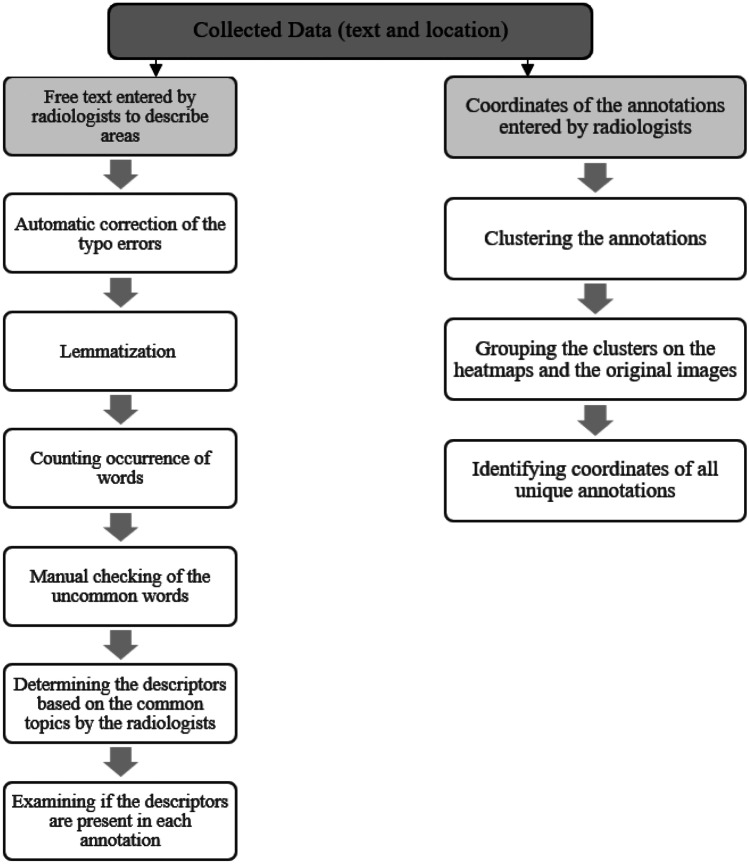
The process of analyzing the recorded annotations from radiologists

For each annotated area across the 20 scans, a free text response entered by the radiologist and the coordinates of a rectangular area selected by the radiologist were saved. As shown in Fig. 2, to analyze the entered text, first an array of tokenized documents was created for each entry. Then, using MATLAB’s *correctSpelling* function, the spelling of the entry was corrected. Stop words were then removed from the array of documents using *removeStopWords* function. This was followed by lemmatization to normalize the text and reduce words to their dictionary forms, known as lemma. For each word, the occurrence was found. All uncommon words were manually checked and words with identical meanings (e.g., small, tiny, < 5 mm) were combined. Finally, all topics from the annotations were identified. Figure 2 shows a flow chart for the process of analyzing the recorded annotations from the radiologists.

For each case, the coordinates for the upper left side of the rectangles were spatially clustered based on the distance between the points. As the left upper point of the rectangle was the first place selected by the radiologists, we found clustering the annotations based on this method (instead of center of the rectangles) resulted in clusters with the most similarity in the provided descriptions. Each point is clustered with the closest neighboring point if the distance between two points is less than 50 pixels. The threshold of 50 pixels was found empirically. We swept all threshold values ranging from 10 to 100 pixels with a step of 10 and found that total number of clusters reduces up to 50 pixels and after that remained unchanged. From 20 cases, 13 cases had 2 more clusters. We manually checked these clusters ensured they represented various areas within the image (e.g., one showing a nodule while the other one is corresponding to an area labeled as vein by more than half of the readers). Geometric median was used to calculate distance to add new points to an existing cluster.

### Data Analysis

Areas on the axial slice were annotated by the radiologists using a range of radiological lexicon, distinguishing pathology (nodules, opacities) and normal structures (such as arteries and veins). We first investigated the percentage of participants who agreed on these high-level labels.

For each extracted clusters, the topics of the annotations were analyzed and the percentage of text entries referring to each topic were found. Topics referring to the nodule characteristics were categorized in two sets [27–30]. The first set contained descriptors associated with a lower likelihood of a nodule being malignant, while the second set were suggestive of a higher malignancy likelihood. Using the Mann–Whitney *U*-test, the percentage of annotations for each set of these topics were compared between benign cases correctly classified as benign by the AI tool, and benign cases misclassified as malignant by the AI tool. Similar analysis was conducted to compare the topics assigned to the malignant cases correctly classified as malignant by the AI tool and malignant cases misclassified as benign by the tool. Finally, the descriptors assigned to the benign cases were compared with the descriptors assigned to the malignant cases.

## Results

**Fig. 3:**
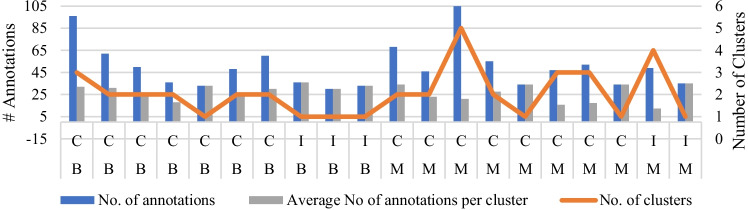
Total number (No.) of annotations, recorded for each case (each point on *x*-axis) and corresponding clustering result. “C” represents the cases, correctly classified by the AI tool while “I” shows the incorrect classification. “M” and “B” represent the malignant and benign cases

In total, 1009 annotations were collected from 36 radiologists. The breakdown of the annotations per case is shown in Fig. 3. In total, radiologists annotated 17 and 24 unique areas on benign and malignant cases, respectively.

### Analysis of Benign Cases

**Fig. 4:**
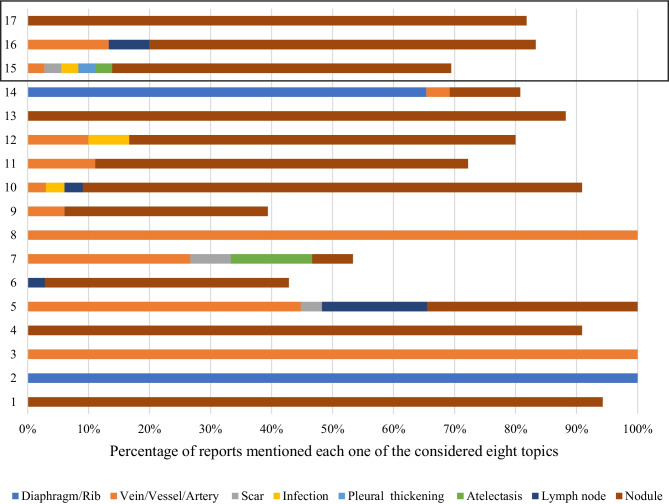
Seventeen areas obtained after clustering the annotations on the benign cases. The last three areas (boxed) were annotated on the benign cases where the AI had misclassified the case as malignant

The results for eight extracted topics (or radiological labels) are shown in Fig. 4. The *y*-axis shows the annotations while the *x*-axis shows the percentage of the annotations mentioned that topic. Overall, the agreement rate among all radiologists was higher for correctly identified benign cases (72% vs 66%, *p* = 0.36). Almost perfect agreement was found for the diaphragmatic/rib area and two vessel/vein areas. The majority of the radiologists annotated misclassified benign cases as nodules (*n* = 20 for label 15, *n* = 19 for label 16, and *n* = 27 for label 17).

Table 1 shows the first and second set of the topics used for describing the areas annotated as nodule by at least one radiologist. The terms in the first set (left sided column) are associated with a lower likelihood of being malignant. Three last items belong to the misclassified cases. For each topic the *p*-value for comparing the correctly classified cases versus misclassified cases are also shown. None of the comparisons yielded a significant *p*-value. The second set of topics used for labeling the areas is also shown in Table 1 in the right column. As stated, these topics are associated with a higher likelihood of being malignant. As shown, the percentages for adenocarcinoma, satellite/spiculated, indeterminate, or lobulated differed significantly among areas correctly classified and misclassified benign cases. This highlights the inherent difficulty of benign case. It implies that only by presenting the areas, significantly important for the decision made by the AI, some of these FPs (misclassified benign cases) cannot be ruled out by radiologist as our as readers more often used terms associated with a higher likelihood of being malignant for these cases.

**Table 1:** For each nodular area on the benign case, the percentage of annotations, containing the first and second set of topics, which are associated with a lower and higher likelihood of a case being malignant, are shown. The bold items are three areas, belonging to the benign cases misclassified as malignant by the tool

	1st set (lower likelihood of malignancy)						2nd set (higher likelihood of malignancy)							
	Solid	Single	Small	Benign	Circumscribed	Calcified granuloma/calcification	Adenocarcinoma	Malignant	Irregular/ill-defined	Satellite/spiculated	Consolidation	Indeterminate	Lobulated	Non-specific
*1*	**69**	**63**	31	3	3	6	0	0	0	0	0	0	0	0
*2*	**52**	**21**	21	15	0	6	0	0	6	0	0	0	0	0
*3*	**14**	**3**	7	0	0	3	0	3	0	3	0	0	0	0
*4*	**6**	**6**	9	9	6	63	0	0	0	0	0	0	0	0
*5*	**0**	**0**	0	0	13	0	0	0	0	0	0	0	0	0
*6*	**3**	**3**	12	9	6	64	0	0	0	0	0	0	0	0
*7*	**36**	**6**	21	6	12	27	0	0	0	0	0	0	0	3
*8*	**17**	**0**	61	6	0	0	0	6	0	0	0	0	0	11
*9*	**17**	**3**	47	7	0	7	0	3	0	0	0	0	0	3
*10*	**32**	**3**	44	9	0	15	0	0	0	0	0	0	0	0
*11*	**4**	**0**	4	4	0	0	0	0	0	0	0	0	0	0
***12***	**22**	**0**	0	3	0	0	8	11	0	6	6	3	3	3
***13***	**33**	**3**	13	7	0	7	0	0	3	0	0	3	0	3
***14***	**48**	**6**	3	9	9	6	3	3	0	3	0	3	3	6
***P***	**0.24**	**0.75**	0.12	0.87	0.8	0.58	** < 0.01**	0.17	0.37	**0.03**	0.06	** < 0.01**	** < 0.01**	**0.04**

### Analysis of Malignant Cases

**Fig. 5:**
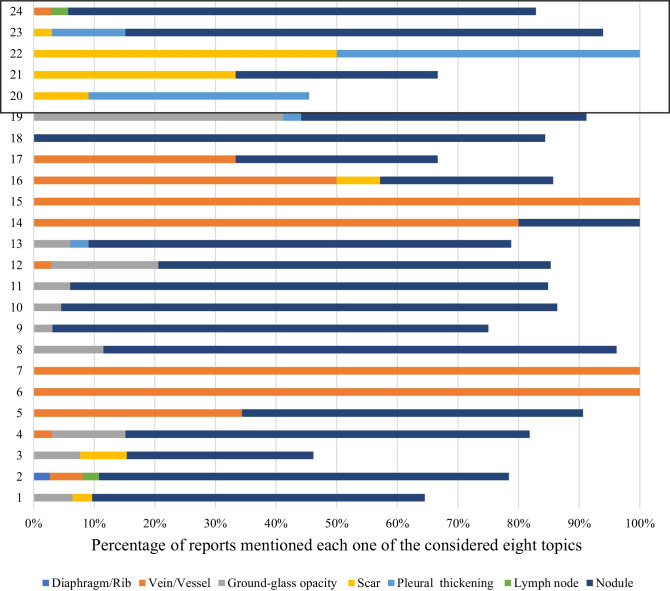
Twenty-four areas obtained after clustering the annotations on the malignant cases. The last five areas were misclassified as benign by the tool

Similar to the abovementioned analysis for the benign cases, 24 areas on the malignant cases were identified (Fig. 5). Overall, the agreement rate among all radiologists was higher for correctly identified cases (70% vs 50%) but, similar to the benign cases, the difference was not significant (*p* = 0.16). Almost perfect agreement was found for three vessel/vein areas.

For each nodule area on the malignant cases, the percentage of annotations containing the topic, associated with a lower (first set) and higher (second set) likelihood of being malignant, are shown in Table 2. As shown, none of the *p*-values for comparing the correctly classified cases to the misclassified was significant.

**Table 2:** For each nodular area on the malignant case, the percentage of annotations, containing the first and second set of topics, which are associated with a lower and higher likelihood of a case being malignant, are shown. The bold items are three areas, belonging to the benign cases misclassified as malignant by the tool

	1st set (lower likelihood of malignancy)						2nd set (higher likelihood of malignancy)							
	Solid	Single	Small	Benign	Circumscribed	Calcified granuloma/calcification	Adenocarcinoma	Malignant	Irregular/ill-defined	Satellite/spiculated	Consolidation	Indeterminate	Lobulated	Non-specific
1	16	0	6	3	3	16	0	19	19	16	3	0	0	0
2	19	0	5	3	0	19	3	8	30	5	5	0	0	0
3	23	0	0	0	0	23	8	8	8	8	15	0	0	0
4	18	0	6	3	0	18	0	18	18	0	0	0	3	0
5	9	6	22	3	6	9	0	6	0	6	0	3	0	6
6	15	8	38	8	0	15	0	12	0	8	0	4	0	8
7	16	0	0	0	0	16	6	22	19	16	3	0	0	0
8	5	5	41	9	0	5	0	5	0	5	0	5	0	9
9	27	0	6	0	0	27	6	27	18	18	3	0	12	0
10	26	0	6	0	0	26	6	18	9	18	0	0	0	0
11	21	0	3	6	0	21	3	18	12	15	3	0	0	0
12	0	0	10	0	0	0	0	0	0	0	0	0	0	0
13	0	0	7	0	0	0	0	0	0	14	0	0	0	7
14	0	0	17	0	0	0	0	17	0	0	0	0	0	17
15	22	0	3	0	0	22	3	22	22	38	3	0	16	0
16	29	0	9	3	0	29	9	21	12	9	3	0	3	0
17	0	0	0	0	0	0	0	9	9	0	0	0	0	0
**18**	0	0	0	0	33	0	0	0	0	0	0	0	0	0
**19**	24	6	15	3	0	24	0	9	15	12	0	0	0	0
**20**	17	3	14	9	0	17	3	17	11	34	0	3	3	0
***P***	0.96	0.14	0.83	0.46	0.27	0.96	0.45	0.34	0.79	0.79	0.15	0.71	0.94	0.30

### Benign Versus Malignant

We also compared the topic of the annotations, provided to the benign cases with the annotations provided to the malignant cases (Table 3). For annotations on benign cases, radiologists used “solid,” “single,” “small,” “benign,” “circumscribed” labels more often when compared to a similar mapped area of a malignant case. On the other hand, “adenocarcinoma,” “malignant,” “irregular/ill-defined,” “satellite/spiculated,” “consolidation,” and “lobulated” were used more on malignant cases. The difference was significant for “single,” “benign,” “malignant,” “irregular/ill-defined,” and “satellite/spiculated” labels. We also compared the topics between misclassified malignant cases with correctly classified benign cases. These two categories of cases were labeled as benign by the AI. We explored if radiologists’ descriptors could hint of AI errors for these cases. Three of the descriptors differed in these two categories. For misclassified benign cases and correctly classified malignant cases, five topics yielded significant or marginally significant *p*-values. Finally, between the misclassified benign cases and the correctly classified malignant cases (i.e., all cases marked as abnormal by AI), the frequency of using “solid,” “circumscribed,” and “indeterminate” differed significantly.

**Table 3:** Average percentage of annotations containing each topic for malignant and benign cases. Areas containing nodules were included. The significant and marginally significant *p*-values are shown using “*.” “B” and “M” represent all of the benign and malignant cases while cB and cM represent correctly classified benign and malignant cases. mB and mM represent misclassified benign and malignant cases

	Malignant	Benign	*p*-value (*B* vs *M*)	*p*-value (cB vs mM)	*p*-value (mB vs cM)
*Solid*	14%	**25%**	0.1553	0.639	0.026*
*Single*	1%	**8%**	0.0186*	0.746	0.126
*Small*	10%	**20%**	0.1824	0.349	0.455
*Adenocarcinoma*	**2%**	1%	0.0770	0.056*	0.569
*Malignant*	**13%**	2%	0.0002*	0.084	0.10
*Benign*	2%	**6%**	0.0099*	0.476	0.268
*Irregular/ill-defined*	**10%**	1%	0.0012*	0.023*	0.139
*Satellite/spiculated*	**11%**	1%	0.0004*	0.023*	0.165
*Circumscribed*	2%	**4%**	0.0867	0.931	0.049*
*Consolidation*	**2%**	0%	0.0526*	0.99	0.953
*Indeterminate*	1%	1%	0.9605	0.056*	0.018*
*Lobulated*	**2%**	0%	0.4282	0.056*	0.237
*Non-specific*	2%	2%	0.5551	0.329	0.188

## Discussion

Deep learning–based AI models for diagnosing a medical image are usually trained to classify cases in two or more categories. To explain these models, various techniques such as class activation mapping and OBSMs can be used.

This study explored if OBSMs can be used to explain the AI decision-making process to radiologists. These maps have been used to identify the areas related to the decision made by the AI in various applications [11, 31, 32]. However, it is unknown whether highlighting an area as an important region without any further labels describing the radiological features of the area, is of any advantage to the radiologists when making their decisions. Considering the complexity of the medical images, there is a chance that different radiologists interpret the highlighted areas on the maps differently. Based on the data presented in this study, the level of agreement on the main radiological label (such as a vessel or nodule) between all radiologists who annotated an OBSM area was very low (only in 12% of all areas (5 out of 41)). Therefore, studies that show how matching radiology descriptors to the mapped areas by an AI could maximize the benefit of that AI tool for the radiologist when thinking about how AI and radiologists can work together. One technique could be to use currently available radiology reports and natural language processing (NLP) techniques to match the saliency maps from AI to radiology descriptors.

Comparison of the topics given to the malignant cases and benign cases showed relevance between the topic and the actual label of the cases. Within the malignant category, the descriptors radiologists used for correctly classified malignant cases did not differ significantly from the descriptors used for misclassified malignant cases. This was a promising result as it implies that based on the original image and presented OBSM, radiologists identified malignant features in misclassified malignant cases, and they might be capable of disregarding, or not being influenced, by an incorrect AI’s output when a true-positive finding is presented. We base this conclusion on the premise that even when radiologist knew the AI misclassified the case and presented an OBSM that may not represent a correct choice, the radiologists still labeled their chosen area with terms that are representative of a malignant diagnosis.

Within the benign category, radiologists used descriptors suggestive of malignancy more often on the misclassified benign cases compared to the correctly classified benign cases. This suggests that presence of the AI and the associated OBSM might influence radiologist towards false-positive decisions for such cases. Despite this, the comparison of misclassified benign cases with real malignant cases indicated that on average radiologists used “solid” and “circumscribed” more often in describing misclassified benign cases compared to the correctly classified malignant cases. Both terms are suggestive of a case being benign [27–30]. Therefore, it can be concluded that the descriptors for these cases sit between correctly classified benign cases and correctly classified malignant cases and there was distinction to some extent to both of classifications.

The current study has a number of limitations. Firstly, only 20 cases were shown to the radiologists and only five of these cases were misclassified by the AI tool, or 25 of the cases in the research study. For generalizing the findings of this study, a larger project will be required. Within this study, a large number of participating radiologists compensated for a low case number and provided sufficient statistical power for our analysis. However, in future works, more cases with more diverse malignant and benign characteristics should be included. Presenting a single slice of the CT scan is another limitation of the current study. Volumetric information is critical in many cases for identifying and describing different elements of the images. Lack of such information could cause discrepancies observed here among the readers. As a future avenue for extending current study, volumetric OBSMs can be explored. Additionally, the recruited radiologists for this study were aware of the actual “decision” assigned to the case by the AI. This could cause some bias in terms of the radiological labels selected by the radiologists, who may tend to use descriptors commonly associated with the benign or malignant nodules. A “pre-and-post” intervention study whereby radiologists label the areas before and after being made aware of the OBSM may assist in our understanding of any biases created by the inclusion of the AI output. This is something that the AI communities and industry will need to develop further in order to gain acceptability with radiology communities. At present, there are very few reviewed studies that present decision-making by radiologists with and without AI prompts and what is the effect on decision-making, perceptual errors, sampling errors, and biases. The laboratory effect [33] could also limit generalizing the finding of the current study to real clinical practice and we do not know whether the recruited readers were familiar with any AI tools and specifically OBSMs.

## Conclusion

Occlusion-based saliency maps represent one technique for interpreting the decision-making processes of an AI system. We investigated the magnitude of agreement among text responses from a group of radiologists to describe areas chosen as diagnostically relevant areas by our AI on LDCT images. Although in several studies some styles of highlighting diagnostically relevant areas have been used to illustrate areas related to the decision made by the AI, it is unknown whether showing them alone, without any further meaningful label, is of any advantage to the radiologists when decision-making. Furthermore, it is not known if the inherent difficulty of identifying these areas would lead to considerable disagreement among radiologists in describing these areas. Our data showed that participating radiologists could only agree on the main radiological label in only 12% of all areas identified.

We also explored whether the recruited radiologists’ descriptions of cases misclassified by the AI provide a rationale for ruling out the AI’s output. The comparison of misclassified benign cases with the correctly classified malignant cases showed that on average radiologists used “solid” and “circumscribed” more often in describing misclassified benign cases compared to the correctly classified malignant cases. Also, differences in the descriptor usage were noted between misclassified malignant cases and correctly classified benign cases and therefore, to some extent, radiologists might be able to rule out these misclassified cases.
